# Biological clocks: their relevance to immune-allergic diseases

**DOI:** 10.1186/s12948-018-0080-0

**Published:** 2018-01-10

**Authors:** Roberto Paganelli, Claudia Petrarca, Mario Di Gioacchino

**Affiliations:** 1 Dipartimento di Medicina e Scienze dell’invecchiamento, Università “G. d’Annunzio” of Chieti-Pescara, Via dei Vestini, 5, 66013 Chieti, Italy; 2 *Ce.S.I.-Me.T., Chieti, Italy

**Keywords:** Circadian rhythm, Biological clock, Immune system, Allergy, Chronopharmacology, Shift work

## Abstract

The 2017 Nobel Prize for Physiology or Medicine, awarded for the discoveries made in the past 15 years on the genetic and molecular mechanisms regulating many physiological functions, has renewed the attention to the importance of circadian rhythms. These originate from a central pacemaker in the suprachiasmatic nucleus in the brain, photoentrained via direct connection with melanopsin containing, intrinsically light-sensitive retinal ganglion cells, and it projects to periphery, thus creating an inner circadian rhythm. This regulates several activities, including sleep, feeding times, energy metabolism, endocrine and immune functions. Disturbances of these rhythms, mainly of wake/sleep, hormonal secretion and feeding, cause decrease in quality of life, as well as being involved in development of obesity, metabolic syndrome and neuropsychiatric disorders. Most immunological functions, from leukocyte numbers, activity and cytokine secretion undergo circadian variations, which might affect susceptibility to infections. The intensity of symptoms and disease severity show a 24 h pattern in many immunological and allergic diseases, including rheumatoid arthritis, bronchial asthma, atopic eczema and chronic urticaria. This is accompanied by altered sleep duration and quality, a major determinant of quality of life. Shift work and travel through time zones as well as artificial light pose new health threats by disrupting the circadian rhythms. Finally, the field of chronopharmacology uses these concepts for delivering drugs in synchrony with biological rhythms.

## Background

The 2017 Nobel Prize for Physiology or Medicine has been awarded to three of the principal scientists who contributed to the discovery of the network of genes and proteins regulating the circadian rhythms based on the light/dark 24 h cycle (“The 2017 Nobel Prize in Physiology or Medicine—Press Release”) [1–3].

Circadian clocks are present in unicellular organisms, in plants, insects and vertebrates [4]. The first gene encoding a critical component of a circadian clock (Period) was discovered in Drosophila by Konopka and Benzer in 1971 [5], showing that circadian clocks are genetically encoded. In mammals, circadian clocks are found in nearly all cells and tissues. They regulate and control physiological processes at the cellular, organ and organismal level, integrating signals received from outside and generated by the normal metabolism. The purpose of different levels of control is to adjust for possible local perturbations, while maintaining a circadian rhythm able to optimize energy allocation for the most likely scenario (which differs during activity and rest periods). For example, the liver clock should be synchronized to rhythms in food intake, but it should also respond to changes in energy demands or variations in oxygen supply. The organization of the mammalian multi-clock system allows for better adaptation to changing environments. This may represent a compromise between flexible adaptation to extremely unpredictable events and circadian stability, which can distinguish also the changes of light–dark hours (and temperature, humidity etc.) with the different seasons [4, 6].

Time keeping signals (“zeitgeber”) in natural conditions are tied to the day-night cycle imposed by the 24 h rotation cycle of the Earth. Light, this very potent zeitgeber, regulates the 24 h sleep–wake rhythm. Sleep precludes both food intake and locomotor activity. Thus, the sleep–wake rhythm governed by sunlight indirectly drives food intake and body temperature cycles. However light and food can be uncoupled (e.g. in the case of jetlag or when food intake is restricted to the natural sleeping phase as in shift work), causing misalignment of these clocks with the daily light–dark cycle of our environment [7, 8]. The field was stimulated by the finding of an intrinsically photosensitive small subgroup of retinal ganglion cells which regulate the circadian rhythms on the light–dark cycle [9, 10] and project to the suprachiasmatic nuclei (SCN) [11], the non-visual brain centers where the mammalian master biological clock is located; this has prompted the search for the molecular clock(s) driving this essential component of all living organisms. A handful of genes and proteins accounting for this complex regulatory central network has been identified.

## Biological clocks

The mammalian core molecular clock consists of two feedback loops [12] connected by a central pair of transcription factors which regulate reciprocally to induce the rhythm of gene expression. The mammalian circadian clock fundamentally depends on two master genes (CLOCK and BMAL1) to drive gene expression and regulate biological functions [6]. CLOCK:BMAL1 heterodimers promote rhythmic chromatin opening and this mediates the binding of other transcription factors adjacent to CLOCK:BMAL1 [13]. Among their targets there is a group of regulatory proteins [PERIOD (PER1, 2 and 3), CRYPTOCHROME (CRY1 and 2), REV-ERB (REV-ERBa and b) and RAR-Related Orphan Receptor (RORa, b and c)]; REV-ERBs and RORs regulate BMAL1 transcription, whereas PER and CRY dimerize to inhibit the BMAL1–CLOCK dimer. PER, the protein encoded by *period* [14, 15] accumulates during the night and is degraded during the day, while other components allow nuclear translocation of PER [16, 17]. Both sleep–wake cycles and many 24-h rhythms persist in the absence of environmental cues and are controlled by internal molecular clocks [18].

Several loops dictate the production of these proteins, including steps of acetylation and phosphorylation, as well as secondary clock-regulated genes which can also feed back on central clock genes [6, 19]. In fact many different organs and tissues express functional molecular clock circuits [20]. None of the mammalian clock components is directly photoreceptive; instead, light signals from the retina are transmitted neuronally to transcription factors that regulate *period* expression. Transcriptional feedback loops are central to the generation and maintenance of circadian rhythms [21, 22]. Clocks in peripheral tissues use essentially the same molecular components as in the SCN; clocks have been detected in different hematopoietic cell lineages, including macrophages and lymphocytes [22, 23].

## The origin of circadian rhythms

In humans, circadian rhythms of 24 h must be synchronized to coincide with the daily rotational cycle of the earth. The alignment of this autonomous circadian rhythm to an external rhythm is defined as entrainment. The light patterns represent the principal environmental stimulus for the rest/activity and sleep/wake cycles [24]. It is also indirectly responsible for timing of food intake, another powerful entrainer of rhythm [24, 25].

**Fig. 1:**
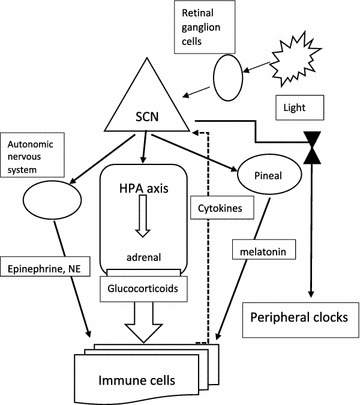
Schematic representation of the master clock regulation of the immune system. Entrainment of the suprachiasmatic nuclei (SCN) is mediated by the input from intrinsically photosensitive retinal ganglion cells activated by light (from the sun and artificial lights from screens and indoor illumination). SCN controls directly the hypothalamus and the hypothalamus–pituitary–adrenal gland (HPA) axis, the autonomous nervous system and the pineal gland. Hormones and neurotransmitters (in boxes) from these clock-regulated structures modulate the activation and functions of different cell types of both the innate and adaptive immune system. Cytokines and chemokines produced by immune cells feed back on the SCN (dotted line). Through transcriptional mechanisms the SCN indirectly regulates also the synchronization of secondary clocks in peripheral tissues and other circadian cycles (wake/sleep, fast/feeding, etc.). *NE* norepinephrine

Circadian photoentrainment is the process by which the internal clock in the deep brain becomes synchronized with the daily external cycle of solar light and dark [4, 9, 26]. The clocks in most mammalian cells are not directly photoreceptive, unlike those of most other organisms, but instead are entrained indirectly to the environmental light–dark cycle via photoreception in the retina, the retino-hypothalamic tract, and a central pacemaker tissue in the suprachiasmatic nucleus (SCN) of the hypothalamus [19]. This process is initiated by a type of retinal ganglion cells that send axonal projections to the SCN, the region of the circadian pacemaker (Fig. 1). In contrast to retinal cells mediating vision, these cells are intrinsically sensitive to light, independent of synaptic input from rod and cone photoreceptors [27]. Photoentrainment of the master pacemaker needs signaling from retinal ganglion cells containing the photopigment melanopsin and intrinsically photosensitive [10]. The cryptochrome/photolyase family of photoreceptors mediates adaptive responses to ultraviolet and blue light exposure in all life forms [28]. The SCN subsequently synchronizes peripheral clocks via mediators including hormones and neuronal signals, primarily using the hypothalamic–pituitary–adrenal (HPA) axis and the autonomic nervous system [6]. The principal hormones i.e. glucocorticoids and catecholamines (epinephrine and norepinephrine), are released by the adrenal gland via the HPA axis [29], but norepinephrine is also derived from sympathetic nerve endings. The HPA is controlled by the SCN which projects to the paraventricular nucleus of the hypothalamus, and this in turn induces the release of adrenocorticotropic hormone by the pituitary, thus regulating the adrenal gland [20, 30]. Catecholamines act via adrenergic receptors, which have many effects on immune cells, as well as increasing the humoral immune responses [24].

## The integrated circadian system

The central biological CLOCK system, influenced by light/dark changes, ‘creates’ the internal circadian rhythms, and the organism ‘feels’ these changes to put in frame physical activities, including energy metabolism, sleep, and immune function.

A recent review [31] listed the following pathological conditions showing diurnal or 24 h patterning, by the organ/tissue/system affected, skin: atopic dermatitis, urticaria, psoriasis, and palmar hyperhidrosis; gastrointestinal: esophageal reflux, peptic ulcer, biliary colic, hepatic variceal hemorrhage, and proctalgia; infection: susceptibility, fever, and mortality; neural: frontal, parietal, temporal, and occipital lobe seizures, Parkinson’s and Alzheimer’s disease, hereditary progressive dystonia, and pain (cancer, post-surgical, diabetic neuropathy, burning mouth and temporomandibular syndromes, fibromyalgia, sciatalgia, and migraine, headache); renal: colic and nocturnal enuresis and polyuria; ocular: conjunctival redness, keratoconjunctivitis sicca, intraocular pressure, anterior ischemic optic neuropathy, and recurrent corneal erosion syndrome; psychiatric/behavioral: major and seasonal affective depressive disorders, bipolar disorder, suicide, and addictive alcohol, tobacco, and heroin cravings and withdrawal phenomena; plus autoimmune and musculoskeletal: rheumatoid arthritis, osteoarthritis, axial spondylarthritides, gout, Sjogren’s syndrome, and systemic lupus erythematosus. Some are directly linked to disruption of circadian rhythms, others result in disturbed sleep with loss of rhythmicity; the peripheral clocks in different tissues become out of phase with the central regulator and other physiologic functions, and this in turn aggravates the symptoms and alters the clinical picture.

## Relevance to immunological functions

A wide range of immune parameters, such as the number of peripheral blood mononuclear cells as well as the level of cytokines, undergo daily fluctuations [32]. Total numbers of hematopoietic stem cells and most mature leukocytes peak in the circulation during the resting phase (during the night for humans) and decrease during the day [24]. Most immune cells express circadian clock genes and present a wide array of genes expressed with a 24-h rhythm. In addition to their functions in the cellular clock, circadian oscillators also participate in the development and specification of immune cell lineages. This has profound impacts on cellular functions, including a daily rhythm in the synthesis and release of cytokines, chemokines and cytolytic factors, the daily gating of the response occurring through pattern recognition receptors, circadian rhythms of cellular functions such as phagocytosis, migration to inflamed or infected tissue, cytolytic activity, and proliferative response to antigens [33]. A pioneering contribution to this area was made by Halberg [34] who discovered a diurnal susceptibility pattern in mice challenged with bacterial endotoxin. The migration of hematopoietic cells to tissues preferentially occurs during the daytime, directed by the circadian expression of cell adhesion molecules and chemokines. During the active phase it is more likely to encounter and detect pathogens and leukocyte trafficking into tissues occurs at the beginning of this phase (early morning). The increased cytokine release at this time point therefore may exacerbate any ongoing local inflammation [24]. One of the mechanisms through which the central clock entrains peripheral tissues is by the production of glucocorticoids in the adrenal gland. Many other circadian signal transduction mediators also regulate the immune response, as melatonin and the autonomic nervous system (Fig. 1).

Perturbation of the redox rhythm (linked to the circadian clock) induced by pathogen challenge triggers immune defense genes without compromising the circadian clock [35]. Activation of innate immunity via TLR4 induces systemic inflammation by eliciting neuroendocrine and leukocyte transcriptional responses, which are regulated by the circadian clock, imposing diurnal rhythm of the inflammatory response [36]. The central clock is sensitive to immune challenge and the brain receives inflammatory signals from the periphery in response to injury/infection. This in turn is thought to exacerbate sickness, develop symptoms like depression, and impair diurnal rhythms of temperature and melatonin secretion [36]. Melatonin, secreted by the pineal gland under SCN control, plays an important role in immune regulation; pinealectomy causes extensive immunosuppression, likely mediated by the decrease in lymphocytes and cytokines such as IL-2, IL-12, and TNF-α [35].

## Sleep and light influences

The time and duration of sleep is tightly controlled by central mechanisms. These may be disrupted by disease processes, but also by other external conditions, such as night shifts, long range flight travels (jet-lag) and social nocturnal activity (social jet-lag). Pro-inflammatory cytokines are generally indicated as sleep-inducing, and basal plasma levels of these cytokines appear higher during the rest phase. Infection-associated sleepiness has been attributed to increased pro-inflammatory cytokine plasma levels [6].

Long-term sleep restriction leads to a gradual increase of circulating leukocytes and subpopulations (neutrophils, monocytes and lymphocytes) with alterations of the number and rhythm of neutrophils persisting after 1 week recovery of sleep [37]; also absolute sleep deprivation alters the rhythmicity of granulocytes [38]. Sleep disorders are one of the most common symptoms in patients with HIV/AIDS [39], but despite the circadian rhythm alteration induced by tat [40] HIV-infected patients with higher HIV Tat protein concentrations had better sleep quality, probably because it increases melatonin production, thus counteracting poor sleep quality induced by HIV [41]. On the opposite spectrum of sleep disorders, narcolepsy, which is generally considered an immune-mediated neurological disease characterized by excessive daytime sleepiness, has been recently characterized by increased inflammatory cytokine production and B and T cell activation markers [42] at variance with other hypersomnia patients who were immunologically distinct and did not present increased plasma cytokines. Many immunological functions depend on the influence of sleep on circadian rhythms, and loss of sleep, in turn, alters the production of glucocorticoids during the night [43]. The neuroendocrine immune response of the HPA axis and sympathetic nervous system, which is activated in response to an antigenic challenge, with a transient inflammatory activity, can lead to metabolic diseases when chronically activated [44], since in all inflammatory conditions high amounts of energy have to be provided for the activated immune system. Experimental animal models and epidemiological data indicate that chronic circadian rhythm disruption increases the risk of metabolic diseases [8].

In patients with rheumatoid arthritis (RA), inflammation is an important covariate for the crosstalk of sleep and the HPA axis. Moreover the interrelation between sleep parameters, inflammation as objectified by C-reactive protein and serum cortisol and adrenocorticotropic hormone levels [45]. Knowledge of circadian rhythms and the influence of glucocorticoids in rheumatology is important [46]: beside optimizing treatment for the core symptoms (e.g. morning stiffness in RA), chronotherapy might also relieve important comorbid conditions such as depression and sleep disturbances [47]. Sleep and circadian disturbances are a frequent complaint of Alzheimer’s disease patients, appearing early in the course of disease, and disruption of many circadian rhythms are present also in Parkinson’s disease [48].

Physiological studies show that aging affects both sleep quality and quantity in humans, and sleep complaints increase with age [49]. Moreover, also feeding/fasting rhythms are compromised.

Circadian expression of secreted signaling molecules transmits timing information between cells and tissues. Such daily rhythms optimize energy use and temporally segregate incompatible processes.

Patients suffering from neuropsychiatric disorders often exhibit a loss of regulation of their biological rhythms which leads to alterations of sleep/wake, feeding, body temperature and hormonal rhythms. Increasing evidence indicates that the circadian system may be directly involved in the etiology of these disorders [50].

Light, especially short wavelength blue light, is the most potent environmental cue in circadian photoentrainment and lens aging is thought to influence this event by acting as a filter for shorter blue wavelengths [51]; light conditions during indoor activities as well as sunlight exposure are of paramount importance to preserve the circadian rhythmicity and avoid a risk factor for several chronic diseases. These considerations impact on the comorbidities of aged subjects, and the importance of the choice of the differential light-filtering properties of intraocular lenses after cataract removal [52].

## Biological clocks and allergic diseases

As an important addendum to the many health consequences of abnormalities of the integrated circadian rhythms, one must just mention disorders in glucose and lipid metabolism as inducers of obesity and the development of Type 2 diabetes [53] and the multifaceted effects of the circadian control of the immune system and its activation [6, 24]. These findings highlight an integrative role of circadian rhythms in physiology [7].

Most studies have dealt with asthma, where symptoms undergo circadian variations with exacerbations occurring more frequently at night (Table 1). Nocturnal asthma is a common presentation and is associated with a more severe form of the disease [54]. Airway diseases are associated with abnormal circadian rhythms of lung function, reflected in daily changes of airway caliber, airway resistance, respiratory symptoms, and abnormal immune-inflammatory responses [55]. The molecular clock is altered by cigarette smoke, LPS, and bacterial and viral infections in mouse and human lungs and in patients with chronic airway diseases. In patients with nocturnal asthma, the difference in FEV1 (and peak expiratory flow rate) between daytime and night may be > 15%. Also diurnal FeNO variation in uncontrolled asthmatics was significantly greater than in controlled asthmatics [56]. Degree of asthma control strongly correlated with sleep quality. Individuals whose asthma was not well controlled took longer to fall asleep, awoke more often, and spent more time awake during the night compared to those with well controlled asthma. Poor asthma control, use of rescue medications, and asthma symptoms were associated with daytime sleepiness and limitations in physical activity and emotional function [57]. In a field study subdividing patients according to preferential time for activity (chronotypes), 35% of asthmatics presented nocturnal symptoms [58] and the morning chronotype was underrepresented when compared to asthmatics without nocturnal symptoms. The timing of drug treatments in asthma (chronotherapeutics) is governed by the circadian nature of asthma. The peripheral clock within the lung is localized in the Clara cell of the mouse bronchial epithelium [59]. Cyclic oscillations in the expression of genes associated with extracellular matrix, cytoskeleton, cell cycle and apoptosis [60], suggest that the repair and turnover of these components in lung are directly or indirectly under the regulation of the lung molecular clock. There is no animal model of asthma with alterations of circadian rhythms due to mutations of components of the molecular clock. However, recently in mice lacking BMAL1 expression in myeloid cells, the induction of asthma caused markedly increased inflammation in the lungs, with higher numbers of eosinophils and increased IL-5 levels in the lung and serum [61]. Moreover, Granulocyte-macrophage colony-stimulating factor mRNA, expressed by activated eosinophils, increased threefold in early morning compared with afternoon in circulating eosinophils from asthma patients with nocturnal symptoms but not in those without [62]. Taken together all data indicate that chronotherapy of asthma offers higher chances of achieving symptoms control, and in particular those developing at night, as in most other allergic diseases [63].

**Table 1:** Circadian rhythm of symptoms in immuno-allergic diseases and acute myocardial infarction as example. Modified from Scheiermann et al. [23]

Disease	Peak time (h)	Symptom	Peak of cytokine/hormone
Asthma	Early morning	Bronchoconstriction	IL5 07 a.m.
Allergic rhinitis	Early morning	Congestion, sneezing	Cortisol 08–11 a.m.
Rheumatoid arthritis	Early morning 05–08 a.m.	Stiffness, pain	TNF+ IL6 06–08 a.m.
Myocardial infarction	Morning 09 a.m.	Pain	Epinephrine + NE 08–11 a.m.

Time when symptoms are more usually presenting is indicated (peak time) and the time of the highest circadian blood level of cytokines and hormones regulated by biological clocks

Also in allergic rhinitis symptoms are commonly most severe during the night or early in the morning, and allergen-induced surface CD203c expression on basophils of seasonal allergic rhinitis patients exhibit a time-of-day-dependent variation [64]. Basophil reactivity shows daily variations depending on the circadian clock activity in basophils, which could partly explain temporal symptomatic variations in allergic rhinitis. The circadian rhythms of salivary melatonin and cortisol were found to be disrupted in patients with allergic rhinitis [65]. Sleep impairment is very common in allergic rhinitis patients and has a significant impact on disease-specific measures of general health and quality of life. The degree of sleep disturbance is directly related to the severity of the disease [66]. Intranasal steroids caused a time shift of PER2 rhythm in the mouse nasal mucosa around the peak of serum glucocorticoids, suggesting that the circadian rhythm of endogenous glucocorticoids regulates the nasal peripheral clock [67] (Table 1). This should imply that in humans steroids should be administered when no time shift can be induced, that is in the early evening.

In the case of atopic eczema, sleep disturbance affects up to 60% of children, rising even higher during exacerbations [68]. This may affect daytime activities and lead to behavioral alterations. The assessment of sleep quality should represent the relevant parameter for control of disease activity, particularly in patients suffering from worsening of symptoms at night [69]. In mice, disruption of biological rhythm causes exacerbation of contact hypersensitivity [70] probably due to altered glucocorticoid rhythmicity. Sleep and daily activity interference are considered important indicators for assessing disease activity and quality of life in chronic spontaneous urticarial [71], and a recent international observational study on quality of life concluded that chronic spontaneous urticaria markedly interfered with sleep and daily activities [72]. Previous studies had revealed circadian variations of histamine levels, peaking at 2 a.m. in mastocytosis [73], but no diurnal changes in basophil numbers [74], although basopenia is often found in chronic urticaria.

It has been shown that the circadian clocks drive the daily rhythms of IgE-mediated allergic reactions in the skin of mice. Also systemic anaphylactic reactions show a diurnal variation, which relies on the circadian clocks [75]; briefly, the circadian clock is a potent regulator the strength of IgE-dependent allergic reactions [63] in the skin but also in other target organs. A mechanistic link is still missing, whereas experimental findings suggest that Clock is a regulator of psoriasis-like skin inflammation in mice via direct modulation of IL-23R [76]. Representative examples of circadian rhythms of symptoms and molecular mediators of immune-allergic diseases are shown in Table 1.

## Conclusion

The 2017 Nobel Prize for Physiology or Medicine has focused the attention on the importance of homeostasis and balanced distribution of energy resources ensured by the presence of circadian rhythms. These are centrally controlled by the master clock in the SCN and photoentrained to the light–dark cycle through inputs from melanopsin-containing retinal ganglion cells. The circadian clocks are not built in a rigid top-down scheme, allowing for oscillations of peripheral clocks in different cells and tissues, thus maximizing flexibility and adaptation to changes in the environment and in the organism. At the biochemical level, they consist of coupled feedback loops that establish a self-sustained, adjustable molecular oscillator that controls, via transcriptional programs, a wide spectrum of cellular and organismal processes. Many physiological events, from sleep to feeding, as well as immune responsiveness, are interlinked to the circadian rhythms. Their disruption can have profound effects on physiology, and modern society and way of life puts increasing pressures to push activity and sleep out of sync with circadian rhythmicity, as in working and eating habits [6]. This poses additional threats to health conditions of workers on night shift, or subjected to long distance travel through many time zones (jet-lag) or working in artificial light conditions mimicking solar light, but with the danger deriving from blue-enriched emitting LEDs and LED screens [51]. Finally, the emerging importance of chrono-feeding (to avoid the epidemics of obesity and associated cardio-metabolic disorders) and chronopharmacology impose changes in current standard practices which have little regard for circadian rhythms.

## Abbreviations

AIDS: acquired immunodeficiency syndrome
CD: cluster of differentiation
FeNO: fractional exhaled nitric oxide
FEV: forced expiratory volume
HIV: human immunodeficiency virus
HPA: hypothalamic pituitary adrenal
Ig: immunoglobulin
IL: interleukin
IL-23R: interleukin-23 receptor
LED: light emitting diode
LPS: lipopolysaccharide
NE: norepinephrine
RA: rheumatoid arthritis
SCN: suprachiasmatic nuclei
TNF: tumor necrosis factor
